# Role of cannabidiolic acid or the combination of cannabigerol/cannabidiol in pain modulation and welfare improvement in horses with chronic osteoarthritis

**DOI:** 10.3389/fvets.2024.1496473

**Published:** 2024-12-10

**Authors:** Francesca Aragona, Marco Tabbì, Enrico Gugliandolo, Claudia Giannetto, Fabiola D’Angelo, Francesco Fazio, Claudia Interlandi

**Affiliations:** ^1^Department of Veterinary Sciences, University of Messina, Messina, Italy; ^2^Independent Practitioner, Varese, Italy

**Keywords:** cannabidiolic acid, cannabigerol, cannabidiol, horses, pain, osteoarthritis

## Abstract

Cannabidiol (CBD) is a non-psychotropic cannabinoid obtained from hemp (*Cannabis sativa* L.) used for pain management in companion animals including horses. The present study aimed to evaluate the efficacy of cannabidiolic acid (CBDA) and cannabigerol/cannabidiol oil (CBG/CBD) oral administration in alleviating pain in adult horses affected by chronic osteoarthritis (OA). Twenty-four horses (10 geldings and 14 mares), aged between 11 and 18 years old, were equally divided into two groups. One group received CBDA 15% oil and the other group received CBG/CBD oil (CBG20%-CBD10%) for 14 consecutive days. A standard dose of 0.07 mg/kg was chosen based on the mean body weight of 450 ± 28 kg. Horse Chronic Pain Scale (HCPS) and physiological parameters monitoring heart rate (HR), respiratory rate (RR), arterial blood pressure (systolic arterial pressure- SAP, diastolic arterial pressure- DAP) were assessed before (T0) and every day for the entire administration (T1-T14). Blood samples were collected for the evaluation of complete hemogram, Leukocyte subpopulation identification and counting and leukocyte differentiation antigens CD4 and CD8 at the day before the administration (T0) and every 7 days (T7 and T14). A reduction of HCPS pain scale scores and the number of WBC, monocytes and neutrophils and CD8 was observed with both CBDA and CBG/CBD treatment. No statistical differences were found in the physiological parameters. No subject required rescue analgesia or showed any adverse effects. The results of this study showed that oral administration of both CBDA and CBG/CBD oil may promote pain reduction in adult horses affected by chronic OA.

## 1 Introduction

Osteoarthritis (OA), also known as degenerative joint disease (DJD), is among the most prevalent and debilitating joint diseases in horses, accounting for 60% of lameness and reduced athletic performance. It is a major cause of premature retirement of both companion and performance horses, resulting in significant economic losses in the equestrian sector (1–4). This musculoskeletal disease is characterized by progressive degeneration of the cartilage surfaces, sclerosis of the subchondral bone, formation of osteophytes and fibrosis of the periarticular tissue. Bone and soft tissue changes are associated to varying degrees of inflammation, which promotes disease progression, reduced joint function, discomfort, pain and compromised animal welfare (5–8).

Therapeutic management of OA in horses includes topical treatments, intraarticular injections and systemic analgesics and anti-inflammatory therapies (9). Non-steroidal anti-inflammatory drugs (NSAIDs) are commonly prescribed in equine practice to treat osteoarthritic pain, and among them non-selective COX inhibitors are the most frequently used. Although COX-2 selective and preferential inhibitors have been introduced and are believed to have a better safety profile, they are still scarcely used, probably due to a perceived low therapeutic efficacy, which has not been conclusively supported by existing literature (10). Because of the potential side effects of these drugs, such as anorexia, gastric irritation, peptic ulcer, nephrotoxicity, hepatotoxicity and hematological diseases but also the lack of a critical evaluation of the pharmacokinetics, pharmacodynamics, safety and efficacy, the long-term use of NSAID in horses is still not recommended (11). In addition, conventional analgesic and anti-inflammatory protocols may not always be effective in subjects with chronic pain due to conditions such as OA that trigger phenomena such as central sensitisation and neuropathic pain. Neuropathic pain can result in allodynia, hyperalgesia (exaggerated response to painful stimuli) and impaired motor control, with significant implications for the well-being of affected animals (12, 13). As a result, alternative therapies are constantly being sought and new treatments are often proposed (14).

Cannabinoids are a group of compounds obtained from hemp (*Cannabis sativa* L.), a member of the Cannabaceae family. Cannabidiolic acid (CBDA) and tetrahydrocannabinolic acid (THCA) are the most abundant of the over 100 cannabinoids found in the cannabis plant (*Cannabis sativa* L.). Better known and marketed compounds are cannabidiol (CBD) and tetrahydrocannabinol (THC) which are the decarboxylated form of the prior molecules produced during heat extraction when processing hemp products (15, 16). Unlike THC, CBD, CBDA and THCA have no psychotropic effects and are widely considered highly tolerable with minimal reported adverse effects such as behavioral changes, altered movements, irritability and diarrhea (17–20). Cannabidiol (CBD) is a non-psychotropic cannabinoid that exerts immunomodulatory antihyperalgesic, antinociceptive and anti-inflammatory effects, acting as a non-competitive allosteric antagonist on G protein-coupled receptors, cannabinoid type 1 (CB1) and cannabinoid type 2 (CB2), which are highly expressed in various parts of the central nervous system (21, 22). CB1 receptors located in the brain modulate neurotransmitter release that prevents excessive neuronal activity, resulting in a calming effect and reduced anxiety, but also regulate movement, postural control and sensory perception. CB2 receptors located in the brain and in peripheral lymphoid tissue mediate the release of cytokines from the immune cells, resulting in a reduction of inflammation and pain. Moreover, mu opioid receptors and CB1 are colocalized in the same neurons of superficial dorsal horn of the spinal cord, the first site of synaptic contact for peripheral nociceptive afferents. This explain the significantly improves in analgesia when a combination of cannabinoids and opioids is used compared to opioids alone (12, 13). The therapeutic use of cannabidiol (CBD) is becoming increasingly popular probably due to its perception as a natural treatment among pet owners (23–26). The analgesic properties of cannabinoids (CBD; THC) appear to be related to their lipophilic properties which allow them to easily cross the blood–brain barrier and induce analgesia (26). Several studies conducted in mice and horses, have shown that CBD reduces the production of inflammatory cytokines such as tumor necrosis factor-α (TNFα) which may reduce inflammation in various body tissues and may have a calming and analgesic effect and lessen anxiety in horses (13, 27, 28).

Cannabinoid synthesis begins with CBGA, which serves as the precursor to most other cannabinoids and is converted into tetrahydrocannabinolic acid (Δ9-THCA), CBDA (cannabidiolic acid), Cannabigerol (CBG) and CBCA (cannabicromenic acid). All enzymatically produced cannabinoids are produced as their acidic form and are then decarboxylated by heat to create the “active” form (29).

Cannabigerol (CBG) is a derivative of *Cannabis sativa* whose precursor is the acid form, cannabigerolic acid (CBGA). CBG is considered a partial agonist of CB1 and CB2 receptors and a regulator of endocannabinoid signaling. Previous studies show that CBG has antioxidant, anti-inflammatory, antitumour, anxiolytic, neuroprotective and dermatological properties and has no psychotomimetic effects (29–31).

Cannabidiolic acid (CBDA), the main compound in plant fibers and seed oil, appears to share several pharmacological characteristics with its neutral analog (CBD). These include its effect as a selective inhibitor of cyclooxygenase-2, its ability to activate the Transient Receptor Potential (TRP) channels vanilloid 1 and ankyrin 1 (TRPV1 and TRPA1, respectively) and to antagonize Transient Receptor Potential Cation Channel Subfamily M Member 8 (TRPM8), a receptor activated during pain and inflammatory processes and in cold sensitisation (32). Despite these similarities, the potential beneficial effects of CBDA are still hidden and unexplored.

Potential medical uses of cannabinoids are under investigation in veterinary medicine for multiple diseases such as pain, seizures, and a variety of other disorders in different species (14, 20, 27, 28, 33–38). The use of CBD has been described in multimodal pharmacological treatments for OA in dogs and horses to improve pain scores and quality of life without serious adverse effects, and as an adjunct or potential alternative to conventional pharmaceutical therapy when standard treatments do not provide adequate symptom relief or are contraindicated (13, 18, 23–25, 39, 40). The use of CBD in horses does not appear to produce neurologic, behavioral, gastrointestinal or any of the other side effects of non-steroidal anti-inflammatory drugs (NSAID) and corticosteroids. However, few studies have investigated the potential therapeutic use of CBD in veterinary medicine, and just some of them have demonstrated the efficacy of CBD. No studies have specifically investigated and compared the effects of CBDA and CBG/CBD combination on chronic OA in horses. The aim of the present study was to evaluate the efficacy of oral administration of CBDA 15% or of CBG20%/CBD 10% oil formulation in alleviating pain in adult horses affected by chronic OA free of any further drug treatment. The authors hypothesized that these cannabinoids could enhance the efficacy of an analgesic protocol selected for the treatment of OA-related pain without causing increased side effects.

## 2 Materials and methods

### 2.1 Ethical approval

The experimental protocols and animal husbandry were performed in accordance with the standards of the Guide for the Care and Use of Laboratory Animals and Directive 2010/63/EU on animal experimentation. The experimental protocol was reviewed and approved by the Institutional Ethical Committee for Animal Care of the University of Messina (approval no. 089/2022). The owners of the enrolled animals were properly informed and signed a written consent to participate in the study.

### 2.2 Animals

Twenty-four (*n* = 24) Italian Saddle Horses (10 geldings and 14 mares), aged between 11 and 18 years and with an average body weight of 450 ± 28 kg, were included in the present study. All subjects were housed at the same equine training center (Italy; latitude 38° 7’ N; longitude 13° 22′ E) under natural environmental conditions. At the time of treatment and sampling, the subjects were housed in individual boxes measuring 3.50 × 3.50 m. Subjects were fed three times a day (at 06:00, 12:00 and 19:00) and received a diet of good quality hay at 11 kg/horse with water available *ad libitum*. Four weeks prior to the start of the study, all subjects were manipulated in their natural environment by the study observers to accustom them to their presence and manipulations.

To exclude the presence of co-morbidities, all subjects underwent a baseline examination prior to the start of the experimental protocol (T0), which included a clinical and orthopedic examination, blood sampling for haematochemical profile, and routine parasitological examinations. Visits and imaging were performed in the stables and lasted no longer than 60 min in total.

Otherwise healthy animals with clinical signs of lameness due to OA localized to one or more joints (metacarpophalangeal or metatarsophalangeal), diagnosed by ‘joint locking’ and confirmed by imaging techniques such as radiography or ultrasonography, were included in the study. Radiographic findings and the location of the OA were obtained and assessed by a board-certified radiologist. OA pain was graded as mild by the attending veterinarian using the Horse Chronic Pain Scale (HCPS).

Subjects who were found to have other comorbid OA conditions at baseline (T0) and those who had taken medication or supplements or undergone orthopedic surgery in the previous 4 weeks were excluded from the study.

### 2.3 Study design

Two oral drop formulations were used in the study: one containing 15% CBDA (Green CBDA Oil, Green Ladybug, Udine, Italy) and the other containing a combination of 20% CBG and 10% CBD (Green CBG/CBD Oil, Green Ladybug, Udine, Italy). Both formulations were commercially available and emulsified in a lipid mixture composed of medium-chain triglycerides (MCTs) derived from coconut oil. The certificate of analysis for the product batch used in this study was issued by the Forensic Toxicology Laboratory Section of the Policlinico Umberto I University Hospital. The analysis confirms the compliance of the product with quality control measures, including the assessment of contamination by microbes, mycotoxins, pesticides, heavy metals and solvents. Using commercial software (Microsoft Office Excel 2013; Microsoft Corp, Redmond, WA, USA), enrolled subjects were randomly divided into two groups: one group received CBDA (CBDA group) and the other received the CBG/CBD combination (CBG/CBD group).

Subjects were weighed using a platform scale to determine the appropriate cannabinoids dose based on individual body weight.

In both groups, a dose of 0.07 mg/kg administered by the oral transmucosal route (OTM) every 24 h for 2 weeks was selected based on the average body weight of the subjects (450–428 kg) (23, 41). All subjects were treated by the same operator to ensure consistency of treatment.

Heart rate (HR, beats/min), respiratory rate (RR, breaths/min) and blood pressure [systolic blood pressure (SAP) and diastolic blood pressure (DAP), mmHg] were measured throughout the 14-day study period. Simultaneously, to assess pain and quality of life, three trained and independent observers completed the Horse Chronic Pain Scale (HCPS) daily at the same time. The HCPS is a validated questionnaire consisting of 15 questions assessing various aspects of the horse’s behavior on a numerical rating scale, all questions were scored from 0 (no interference) to 3 (total interference) (42, 43). The total pain score ranged from zero (no pain) to 45 (maximum pain). All questions have been described in previous studies (25).

Both physiological parameter values (HR, RR, SAP and DAP) and HCPS score assessments were collected at the same time (17:00–18:00) starting before treatment initiation (T1, baseline) and every day for 14 days (T14), to avoid data fluctuations due to circadian rhythms (44). Measurements were taken at the following time points: day one (T1, baseline), day two (T2), day three (T3), day four (T4), day five (T5), day six (T6), day seven (T7), day eight (T8), day nine (T9), day ten (T10), day eleven (T11), day twelve (T12), day thirteen (T13), and day fourteen (T14). Each measurement was performed prior to feeding time using a multi-parameter monitor (Datex-Ohmeda S/5; Finland) HR was measured using a pulse oximeter was placed on the upper lip and an occluding cuff (size 7.2/13 cm) was placed on the tail via the oscillometric method. RR was determined by direct observation of chest wall excursions by the same operator (45).

If the HCPS scores increased (score > 12), subjects were excluded from the study and rescue analgesia was administered with intravenous phenylbutazone (Bute 200 mg/mL, ACME, Italy) at a dose of 2.2 mg/kg per day for 5 days. Furthermore, attention was paid to signs such as sneezing, head shaking, licking, nausea, salivation and signs of sedation, lethargy, ataxia, incoordination, increased or decreased appetite, urinary hypersensitivity, incontinence, diarrhea, mydriasis, hypothermia, blepharospasm, photophobia or nystagmus. The occurrence of any of these signs or other adverse effects was assessed through continuous monitoring of the subjects enrolled in the study by the stable staff.

### 2.4 Hematological analysis

Blood samples were collected from all horses immediately following the assessment of physiological parameters and HCPS score, prior to CBDA and CBG/CBD administration, at T1, T7 and T14 data points. Blood was collected by jugular venipuncture into Vacutainer tubes (6 mL) containing ethylenediaminetetraacetic acid (K3 EDTA) (Terumo Corporation, Tokyo, Japan) as anticoagulant in duplicate. A blood smear was immediately made from the EDTA tubes. After air drying, the slides were stored and taken to the laboratory. Blood aliquots were immediately refrigerated at 4°C and then the blood count was assessed (within 3–4 h).

All prepared slides were stained with the May-Grünwald stain, which consists of the successive application of two neutral stains: the May-Grünwald mixture (1902), derived from the Romanowsky mixture (1891), and the Giemsa mixture (1904). In preparations fixed by rapid drying, the basic or acidic nature of the cytoplasm and the granulation of the leucocytes were noted. Microscopic analysis of the blood films was performed using a light microscope (Nikon Eclipse e200 Nikon Instruments Europe BV, Amsterdam, The Netherlands) at 1000× magnification with oil. Leukocyte identification and counting was performed on all samples using a manual 100-cell differential count to identify neutrophils, basophils, eosinophils, lymphocytes, and monocytes on each blood film.

Whole blood was used for the evaluation of the complete hemogram including red blood cells (RBCs), hemoglobin (HGB), hematocrit (HCT), white blood cells (WBCs) and platelets (PLTs) using an automated hematology analyzer (HeCo Vet C. SEAC. Florence. Italy) and used for flow cytometry analysis for the determination of leukocyte differentiation antigens CD4 and CD8. Fluorescence activated cell sorting and analysis was performed using a FACS Attune Nxt (Thermo Fisher Scientific). Briefly 200 μL of whole blood was stained with anti-horse monoclonal antibodies CD4-FITC (Thermo Scientific MA528355) and CD8-PE (Thermo Scientific MA528426) for 45 min and then lysed with BD Pharm Lyse^™^ Lysing Buffer (BD Biosciences). Cells were first gated for lymphocytes and doublet exclusion and then gated for T cell populations by CD4 and CD8.

### 2.5 Statistical analysis

The statistical analysis was performed by statistical software Graph Pad Prism v. 9.5.1 (Graphpad Software Ldt., USA). The assumption of clinical data normality was examined by a Shapiro–Wilk test. Clinical data (HR, RR, SAP and DAP) and scores from Horse Chronic Pain Scale were reported as median and range. Hematological parameters were reported as mean ± standard deviation (SD) of every observation. Differences between groups and data points were performed using a two-way for repeated measure analysis of variance- ANOVA followed by the Bonferroni *post hoc* test for multiple comparisons. When comparing the time points, only those values found to be statistically significant compared to T1 were considered for the following statistical analysis. The scores from Horse Chronic Pain Scale were assigned by three observers unaware of the treatment administered and Kendall’s concordance coefficient W was calculated. *p* value *p* < 0.05 was considered statistically significant.

## 3 Results

Data were not normally distributed. The level of interobserver agreement was high (*W* = 1). The total number of subjects involved in the research study was 24, with an effective power of 0.80.

Physiological values were within the physiological ranges for the species and reported in Table 1. Both groups showed no significant variations among time points compared to T1: HR (*p* = 0.18; *F* = 2.54_(13,52)_); RR (*p* = 0.20; *F* = 1.84_(13,52)_); Syst (*p* = 0.72; *F* = 1.64_(13,52)_); Diast (*p* = 0.42; *F* = 1.03_(13,52)_). No significant variation were found between groups: HR (*p* = 0.10; *F* = 1.98_(1,4)_); RR (*p* = 0.13; *F* = 3.63_(1,4)_); Syst (*p* = 0.54; *F* = 0.01_(1,4)_); Diast (*p* = 0.66; *F* = 0.22_(1,4)_).

**Table 1:** Clinical parameters expressed as median and range.

Groups	Variable	Monitoring days													
		T1	T2	T3	T4	T5	T6	T7	T8	T9	T10	T11	T12	T13	T14
CBDA	HR	32 30/34	32 30/34	32 31/33	33 32/33	34 32/36	31 30/32	30 29/31	30 29/31	32 31/33	32 31/33	31 28.32	32 31/33	30 29/33	32 30/34
	RR	14 12/16	16 12/20	16 12/20	16 12/20	16 12/24	16 12/20	20 16/24	16 12/20	14 12/18	12 12/16	16 12/20	18 12/24	16 12/24	16 12/20
	SAP	94 93/95	94 90/100	98 96/100	100 96/104	100 110/91	108,100/118	108 98/120	100 95/105	95 83/108	102 100/104	100 96/104	97 86/108	93 90/98	93 92/95
	DAP	59 52/68	63 50/78	65 57/70	72 67/77	84 73/96	81 70/94	65 56/74	67 52/82	67 53/81	68 50/84	62 56/68	60 54/66	60 50/72	62 61/63
CBG/CBD	HR	31 29/37	30 29/32	31 29/33	33 32/33	35 29/45	34 31/38	33 27/42	33 30/36	30 2,634	31 27/38	28 26/32	30 26/36	30 28/34	31 29/32
	RR	13 12/16	15 12/20	18 12/24	13 12/16	13 12/16	13 12/16	13 12/16	13 12/16	13 12/16	13 12/16	16 12/16	12 12/16	12 12/16	14 12/20
	SAP	94 90/102	97 89/108	102 97/112	100 89/110	103 92/114	103 94/118	105 80/120	101 94/110	95 84/104	101 81/117	100 89/115	104 95/115	95 95/108	103 88/111
	DAP	63 52/77	64 50/84	63 60/64	69 54/92	79 70/87	75 67/86	74 63/83	73 63/85	71 55/82	72 55/86	65 46/92	67 63/76	67 63/76	64 53/74

Heart rate (HR), respiratory rate (RR), systolic arterial pressure (SAP), diastolic arterial pressure (DAP).

Total pain scores from horses Chronic Pain Scale (HCPS) questions for subjects in each group are shown in Table 2. No difference in scores between the two groups at baseline (T1) (*p* = 0.36; *F* = 1.05_(1,4)_). However, a significant difference between the two groups was observed at T3, T4, T5 and T6 (*p* < 0.05). A reduction in HCPS scores from T3 to T14 in CBDA group (*p* < 0.0001) and from T6 to T14 compared to T1 in CBG/CBD group (*p* < 0.0001) was observed. No subject required rescue analgesia. No adverse effects were observed in any subject.

**Table 2:** Total Composite Pain Score of all horses monitored were expressed with median and range.

Groups	Monitoring days													
	T1	T2	T3	T4	T5	T6	T7	T8	T9	T10	T11	T12	T13	T14
CBDA	10 9/12	6 5/9	5^1^^,*^ 5/8	5^1^^,*^ 5/7	5^1^^,*^ 5/7	5^1^^,*^ 4/7	4^1^ 4/6	4^1^ 4/6	4^1^ 4/6	3^1^ 3/5	3^1^ 3/3	3^1^ 3/3	3^1^ 2/3	3^1^ 2/3
CBG/CBD	11 5/13	10 5/13	9 4/12	9 4/10	8 4/10	8 3/10	6^1^ 3/8	6^1^ 3/8	6^1^ 2/8	5^1^ 2/7	5^1^ 2/7	5^1^ 2/7	5^1^ 2/7	5^1^ 2/6

1 Significant difference within the group at time points compared to baseline (T1).

^*^Significant difference between groups (*p* < 0.05).

**Figure 1:**
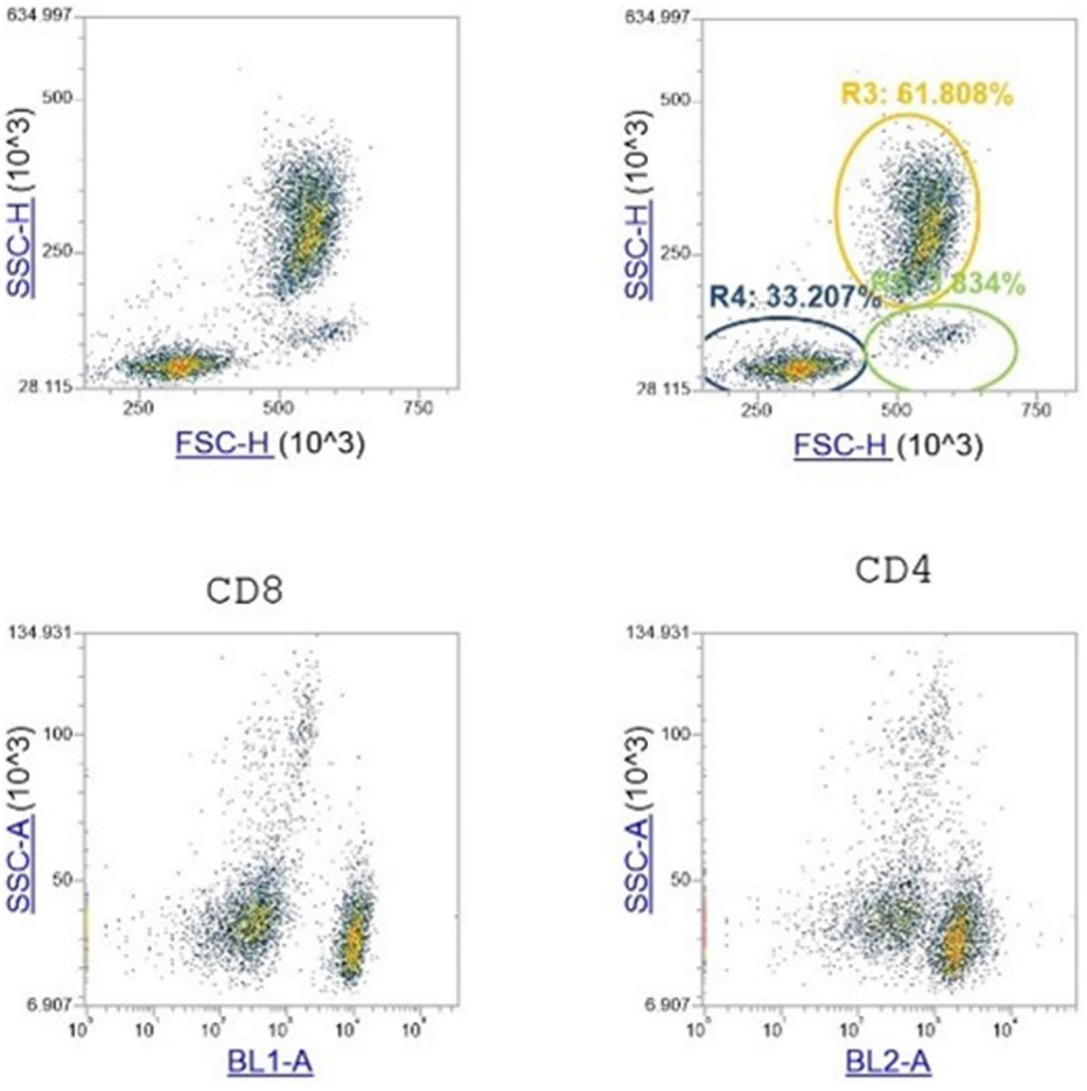
Flow cytometry analysis. Data from the lymphocytes interval gate in the FSC versus SSC scatter plots are shown. The gate-shaded in this plot are the singlet WBCs. Then plotted in the SSC versus BL1 and 2 scatter plot for CD4 and CD8 identification cells. The gate was drawn on the scatter plot where almost all of the lymphocytes lies.

The hematological variables, leukocyte formula and CD4 and CD8 cells trend (Figure 1) observed during the 2- weeks period is shown in Tables 3–5. A significant reduction was observed on WBC count at T3 compared to T1 in both groups (*p* < 0.001), on Monocytes in CBDA group (*p* < 0.001) at T2 and T3 compared to T1, on Neutrophils in CBG/CBD group (*p* < 0.001) at T2 and T3 compared to T1, and on CD8 cells (*p* < 0.01) in CBG/CBD group at T2 and T3 compared to T1.

**Table 3:** White blood cells (WBCs), red blood cells (RBCs), hemoglobin (HGB) and hematocrit (HCT) in two groups monitored at T1, T7 and T14 Data are showed as mean ± SD.

Variables	Groups	Monitoring days		
		T1	T7	T14
WBCs (10^3/Ul)	CBDA	7.2 ± 0.99	6.9 ± 0.85	6.4 ± 0^1^
RBCs (10^6/Ul)		6.72 ± 0.69	6.69 ± 0.74	6.6 ± 0.27
HGB (g/dL)		12.2 ± 2.26	11.8 ± 2.55	11.7 ± 0.28
HCT (%)		35.4 ± 6.79	35.25 ± 6.86	34.5 ± 1.84
WBCs (10^3/Ul)	CBG/CBD	6.68 ± 0.97	6.35 ± 1.22	5.58 ± 0.64^1^
RBCs (10^6/Ul)		6.61 ± 0.39	6.89 ± 0.27	6.91 ± 0.53
HGB (g/dL)		10.98 ± 0.90	11.52 ± 1.40	11.43 ± 1.46
HCT (%)		32.88 ± 2.46	33.85 ± 3.69	34.75 ± 3.49

1*p* < 0.05 vs. Day 1.

1 Significant difference within the group at time points compared to baseline (T1).

**Table 4:** Mean ± SD and significance of CD4 count, CD8 count and CD4/CD8 ratio in CBDA and CBG/CBD groups at T1, T7, T14 expressed in %.

Variables	Groups	Monitoring days		
		T1	T7	T14
CD4 (%)	CBDA	43 ± 12.2	45.5 ± 7.78	47.5 ± 3.54
CD8 (%)		10 ± 2.83	10 ± 0	11.5 ± 0.71
CD4/CD8 ratio		4.6 ± 0.14	4.55 ± 0.78	4.13 ± 0.53
CD4 (%)	CBG/CBD	47.75 ± 6.40	47.5 ± 6.45	47 ± 8.88
CD8 (%)		18.75 ± 4.79	14.5 ± 4.04^1^	13 ± 2.94^1^
CD4/CD8 ratio		2.6 ± 0.62	3.39 ± 0.91	3.73 ± 1.30

1*p* < 0.05 vs. Day 1.

1 Significant difference within the group at time points compared to baseline (T1).

**Table 5:** Mean values ± standard deviation (±SD) of Basophils, eosinophils, monocytes, Neutrophils and Lymphocytes obtained in CBDA and CBG/CBD groups at T1, T7, T14 expressed in %.

Variables	Groups	Monitoring days		
		T1	T7	T14
Basophils (10^3/Ul)	CBDA	0.03 ± 0.04	0.03 ± 0.04	0.06 ± 0.09
Eosinophils (10^3/Ul)		0.07 ± 0.01	0.32 ± 0.18	0.16 ± 0.04
Monocytes (10^3/Ul)		0.4 ± 0.01	0.04 ± 0.05^1^	0.06 ± 0.09^1^
Neutrophils (10^3/Ul)		4.13 ± 0.01	4.18 ± 0.4	3.78 ± 0.27
Lymphocytes (10^3/Ul)		2.70 ± 1.49	2.35 ± 0.93	2.33 ± 0.18
Basophils (10^3/Ul)	CBG/CBD	0.28 ± 0.3	0.05 ± 0.03	0.00 ± 0
Eosinophils (10^3/Ul)		0.03 ± 0.05	0.11 ± 0.09	0.10 ± 0.07
Monocytes (10^3/Ul)		0.27 ± 0.1	0.25 ± 0.08	0.15 ± 0.07
Neutrophils (10^3/Ul)		4.93 ± 0.3	3.77 ± 0.31^1^	3.54 ± 0.4^1^
Lymphocytes (10^3/Ul)		1.67 ± 0.62	2.16 ± 1.17	1.81 ± 0.45

1*p* < 0.05 vs. Day 1.

1 Significant difference within the group at time points compared to baseline (T1).

## 4 Discussion

The results of this study showed that the use of CBDA and the combination of CBG/CBD oil at the dosage administered did not influence physiological and hematological parameters in horses with chronic OA. This contrasts with results obtained in previous studies on horses with acute OA treated with CBD oil, where the same parameters, such as HR and RR, were influenced by pain and decreased significantly after treatment (25). In this study, however, these parameters remained constant throughout the experimental period in both groups. The methods used to establish a direct relationship between changes in physiological parameters and the presence or severity of pain were validated by previous studies (25, 46, 47).

Over a 14-day treatment period, the administration of CBDA and the CBG/CBD combination resulted in stabilized HR, RR, and blood pressure, accompanied by a significant reduction in pain scores. This reduction in pain led to an overall improvement in the clinical condition in both treatment groups. Notably, pain reduction was observed earlier in the CBDA group at T3, compared to the CBG/CBD group, where significant pain relief was evident only from T7 onward. This suggests a potentially quicker onset of action for CBDA, probably thanks to the acid form of CBDA that is available faster than the others.

Our findings align with previous studies that demonstrated the anti-inflammatory and anxiolytic effects of cannabinoids in animal models. For example, CBDA has been shown to exert these effects when administered systemically or orally in rodent studies, both before and after carrageenan-induced inflammation (32). Previous studies have used a mix of CBD and CBDA in dogs with chronic OA administered as a dietary supplement from 4 to 8 weeks and indeed found a reduction in pain (47). Furthermore, Cabrera et al. used a CBD and CBG combination similarly to the present study, demonstrating clear anti-inflammatory effects in the lungs of humans (48). In horses, previous studies have shown a positive effect against stress and anxiety, assessed according to different scales of scores and values, as in our case, considering the effect on HR, which tends to be reduced (49, 50). The use of CBD has shown a reduction in anxiety and stress by monitoring HR also in rodents (51) and humans (52).

In this study, the use of CBDA and CBG/CBD at 0.07 mg/kg led to significant changes in inflammatory markers over time. Specifically, there was a reduction in the number of leukocytes and their subpopulations, including a decrease in CD8+ T cells, neutrophils (in the CBG/CBD group), and monocytes (in the CBDA group). The reduction in the leukocyte population suggests a possible overall reduction in the inflammatory process following treatment with CBDA and CBG/CBD.

These findings are particularly relevant considering the role of immune cells in OA pathogenesis (53). The innate immune system is essential in the host’s defense against microbial invasion and the modulation of various types of tissue injury and repair (54). Immune responses within the joint cavity have regulatory roles in driving cartilage injury toward repair or destruction. Restoration and healing, if they occur, are accompanied by the secretion of anti-inflammatory factors from immune cells and chondrogenesis. However, once damaged, the affected cartilage cannot regenerate itself. In this pathological condition, augmented inflammatory responses develop cartilage injury to OA (55). Macrophages, T cells, natural killer cells, dendritic cells (DCs), and neutrophils are among the immune cells primarily involved in cartilage injury and repair in OA pathophysiology (55). More specifically, T cells and B cells in the synovial fluid of OA patients are regulated by neutrophils, leading to cartilage breakdown and bone remodeling (53). Neutrophils, the most abundant circulating leukocytes, play a crucial role in both joint repair and chronic inflammation. They contribute to tissue repair by phagocytizing necrotic cells, releasing anti-inflammatory factors, and activating protective genes, but they also drive chronic inflammation through enzyme release and immune cell activation (56). The observed reduction in CD8+ T cells at T3 in both treatment groups may indicate a decrease in tissue destruction, as these cells are known for their cytotoxic capabilities, which contribute to tissue damage in various pathophysiological conditions (57).

In addition to the reduction in pain observed in the subjects undergoing treatment, we also noted a reduction in the number of leukocytes and their subpopulations, suggesting a possible overall reduction in the inflammatory process with both CBDA and CBG/CBD treatment (58, 59). The most interesting result, obtained using a dose of 0.07 mg/kg (CBDA 15% or CBG 10%/CBD 20%), is the variation and, in particular, the reduction over time of certain inflammatory markers compared at point T1, such as the number of WBCs in both groups, a reduction of CD8+ cells, a reduction of neutrophils in the CBG group, and a reduction of monocytes in the CBDA group. These responses involving leukocyte populations and subpopulations could be associated with a reduction in the ongoing inflammatory process due to the action of the products administered in both groups.

Despite our results are promising, this study has some limitations, including the small number of animals, the measurement of serum biochemical parameters (liver transaminases) and urine examination. The lack of a control group receiving conventional analgesics to compare with the groups receiving cannabinoids is another limitation of the study. Furthermore, considering other biomarkers of inflammation such as serum pro-inflammatory and anti-inflammatory cytokines should be advisable, as well as exploring the effects of higher cannabinoid doses, depending on the pathophysiological state of the animals. Based on the data collected in this study, the addition of CBDA 15% or CBG10%/CBD 20% in horses with chronic OA resulted in stable HR and RR, reduced pain scale scores, and a decrease in hematological markers of inflammation. These findings appear to be correlated with a reduction of the inflammatory process and an improvement in the quality of life of treated subjects.

## 5 Conclusion

Oral transmucosal administration of 15% CBDA oil and CBG/CBD oil (CBG20%-CBD10%) for 14 consecutive days at a dose of 0.07 mg/kg reduced HCPS scores, and decreased leukocyte, monocyte and neutrophil populations and CD8 markers. The physiological parameters monitored (HR, RR, SAP and DAP) showed no changes throughout the study period. None of the animals required rescue analgesia or experienced any adverse effects. The results support the efficacy of CBDA and CBG/CBD oils in providing optimal pain management and an overall more satisfactory quality of life in adult horses with chronic OA. Further research is required to fully understand the pharmacodynamics and pharmacokinetics of cannabinoids, to establish an optimal dosage range and to determine potential applications in this species.

## Data Availability

The original contributions presented in the study are included in the article/supplementary material, further inquiries can be directed to the corresponding authors.
